# Feature Selection Method Based on Neighborhood Relationships: Applications in EEG Signal Identification and Chinese Character Recognition

**DOI:** 10.3390/s16060871

**Published:** 2016-06-14

**Authors:** Yu-Xiang Zhao, Chien-Hsing Chou

**Affiliations:** 1 Department of Computer Science & Information Engineering, National Quemoy University, 89250 Kinmen Island, Taiwan; 2 Department of Electrical Engineering, Tamkang University, 25137 New Taipei City, Taiwan; chchou@mail.tku.edu.tw

**Keywords:** feature selection, neighborhood relationship, EEG signal, Chinese character recognition

## Abstract

In this study, a new feature selection algorithm, the neighborhood-relationship feature selection (NRFS) algorithm, is proposed for identifying rat electroencephalogram signals and recognizing Chinese characters. In these two applications, dependent relationships exist among the feature vectors and their neighboring feature vectors. Therefore, the proposed NRFS algorithm was designed for solving this problem. By applying the NRFS algorithm, unselected feature vectors have a high priority of being added into the feature subset if the neighboring feature vectors have been selected. In addition, selected feature vectors have a high priority of being eliminated if the neighboring feature vectors are not selected. In the experiments conducted in this study, the NRFS algorithm was compared with two feature algorithms. The experimental results indicated that the NRFS algorithm can extract the crucial frequency bands for identifying rat vigilance states and identifying crucial character regions for recognizing Chinese characters.

## 1. Introduction

Sleep is a physiological state comprising multiple stages. Electroencephalogram (EEG) analysis has indicated that typical patterns of activity are correlated with various stages of sleep, wakefulness, and certain pathophysiological processes, such as seizures. For many researchers, identifying sleep stages is important, for example, sleep stage identification is important sleep deprivation and seizure studies [1,2,3,4,5]. Typically, sleep stages can be identified by combining EEG, electromyography (EMG), electrooculography (EOG), and visual behavioral monitoring. However, scoring these vigilance states manually is a time-consuming task, even when the analyzer is an expert.

The vigilance stages of rats are generally classified as three states [6,7,8,9]: the awake (AW) state, slow wave sleep (SWS) state, and rapid eye movement (REM) sleep state [9]. During the AW state, the rats produced high-frequency EEG results. Several researchers have distinguished active awake from quiet awake based on high EMG activity. The spectrum of EEG in the AW state includes a high-power alpha wave (8–13 Hz) and gamma wave (20–50 Hz). The SWS state, which is defined by a high-amplitude and low-frequency EEG, begins with a sleep spindle and is dominated by a delta (0.5–4 Hz) wave. In the REM state, the rats produced high-frequency EEG results, which were similar to those produced in the AW state. However, the rats were atonic and demonstrated flat EMG activity. Alpha and gamma waves that display high activity are also characteristics of the REM state.

**Figure 1:**
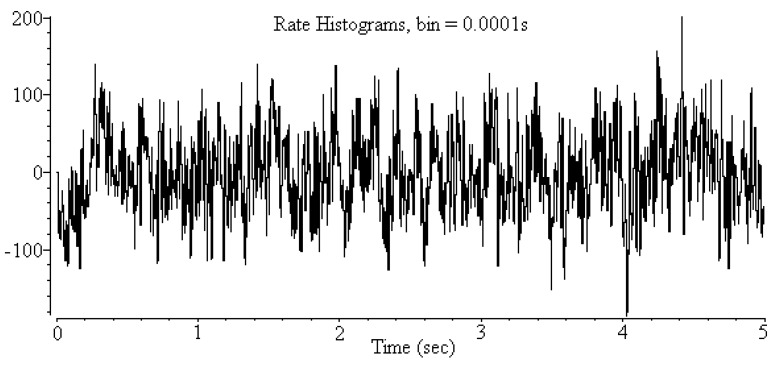
Electroencephalogram (EEG) signal.

**Figure 2:**
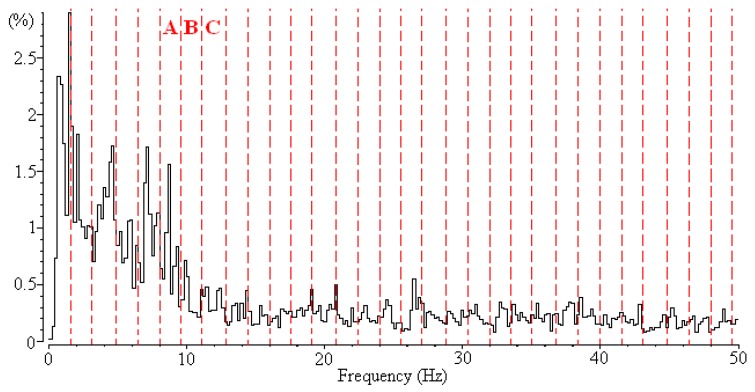
EEG spectrum obtained by applying fast Fourier transform (FFT) and dividing its resolution into 32 frequency bands.

In our previous study [9], we proposed a machine learning method to classify three vigilance stages of rats with a high accuracy rate. However, biological researchers are typically concerned with the crucial frequency bands used to classify these three vigilance states. The intuitive method used to extract crucial frequency bands is applying a feature selection algorithm to extract the features and identify the corresponding frequency bands based on these extracted features. To extract features, the EEG signal is first converted into frequency information by using fast Fourier transform (FFT) [10,11] (Figure 1). The EEG spectrum is generated using the FFT method with a frequency range from 0 to 50 Hz; then the EEG spectrum is uniformly divided into 32 nonoverlapping frequency bands (Figure 2). The frequency range of each frequency band is 1.6 Hz. The power of each frequency band is normalized according to the sum of the power of the frequency bands, and 32 numerical feature vectors are subsequently generated. A feature selection algorithm can then be used to extract a feature subset for classifying vigilance states. Examining the selected feature vectors in the feature subset reveals the crucial frequency bands.

Although a feature selection algorithm can be used to extract the crucial frequency bands of EEG signals, it creates a perplexed situation when applied to this problem. In the data set of EEG signals, each frequency feature vector may have a dependent relationship with neighboring feature vectors. For example (Figure 2), the power of the alpha wave is the main characteristic used to classify vigilance states (e.g., the awake state), and the frequency bands A, B, and C belong to the alpha wave (8–13 Hz). Although these feature vectors (A, B, and C) denote different frequency bands, biological researchers have agreed that assuming that one feature vector is completely independent of the other two feature vectors is unreasonable. For example, if Feature B is selected as a crucial frequency band for identifying EEG signals, most biological researchers would agree that Features A and C are likely to be crucial frequency bands.

**Figure 3:**
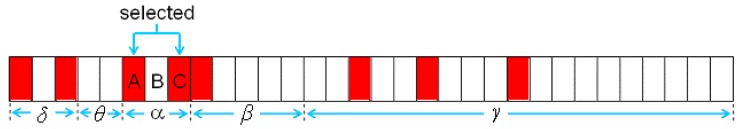
Example indicating that only Features A and C are selected as the crucial frequency bands, whereas Feature B is not selected.

However, most feature selection algorithms are not designed for use in these types of scenarios; therefore, using these algorithms might produce unreasonable selection results. According to one of our simulation results, the information gain (IG) algorithm [12] can be applied as the feature selection algorithm. Figure 3 shows the feature subset selected by the IG algorithm. Eight feature vectors are selected. By examining the alpha wave, only Features A and C are selected by the IG algorithm, whereas Feature B is not selected. This means that the 8–9.6 Hz and 11.2–12.8 Hz frequency bands are important, but the 9.6–11.2 Hz frequency band is irrelevant. However, according the experience of biological researchers, the result makes it difficult to determine whether the alpha wave is the crucial frequency bands for identifying sleep stages.

Regarding Chinese character recognition, a character image is typically normalized first using the nonlinear normalization technique and is then divided into several subimages. For each subimage, a numerical feature vector is obtained by calculating the specified image characteristics of this subimage. Each numerical feature vector represents the image information of the corresponding subimage in a character image. Similar to the feature vector of the EEG-signal data set, each Chinese-character feature vector has a dependent relationship with neighboring feature vectors (additional details are described in Section 4.2). This situation may create more difficulties for applying a feature selection algorithm.

The aforementioned observations were the motivations of this study in which a novel feature selection scheme for EEG signal identification and Chinese character recognition is proposed. In the two applications, dependent relationships exist among the feature vectors and neighboring feature vectors. By applying the proposed algorithm, unselected feature vectors have a high priority of being added into the feature subset if the neighboring feature vectors have been selected. In addition, selected feature vectors have a high priority of being eliminated if neighboring feature vectors are not selected. Additional details are described in Section 3. The remainder of this paper is organized as follows: Section 2 presents a brief review of feature selection algorithms. In Section 3, the proposed feature selection algorithm is presented. Section 4 introduces the method for generating experimental data sets. In Section 5, the experimental results are presented to demonstrate the effectiveness of the proposed algorithm. In Section 6, the discussions of the proposed algorithm are given. Section 7 concludes the paper.

## 2. Brief Review of Feature Selection Algorithms

A successful feature selection algorithm can extract specific features with which users are concerned [13,14,15,16,17]. For example, researchers can identify the genes that may lead to certain diseases by using feature selection algorithms to analyze the microarray data [13]. In analyzing DNA sequences, feature selection algorithms have been applied to locate segments on the sequence or identify types of amino acids [14,15]. Feature selection algorithms also facilitate the extraction of keywords in text classification [16,17].

Researchers have proposed numerous feature selection methods in recent years [18,19,20,21,22,23,24,25,26,27,28,29,30,31,32,33,34]. Guyon and Elisseeff [18] categorized feature selection algorithms as wrappers, filters, and hybrid algorithms. The following discussion provides a brief introduction on these feature selection algorithms.

1. Filters: In the filters method, the importance of the features is ranked according to statistical criteria or information-theoretic criteria [19,20,21,22]. IG or the X2 statistic is typically used to extract the features in text categorization [21,22].
2. Wrappers: The wrappers method involves the extraction of the optimal feature subset by adopting a specific searching strategy and performing continual evaluations [23,24,25,26]. These strategies include the sequential floating search [23], adaptive floating search [24], branch and bound [25], and genetic algorithm [26] methods.
3. Hybrid: The hybrid method extracts several feature subsets by combining both filters and wrappers using an independent feature evaluation method. In addition, the optimal feature subset is extracted by processing the classification algorithm. This strategy is performed repeatedly until obtaining any more favorable feature subsets is not possible [27,28].

Information Gain (IG) [12] is a general feature selection algorithm for evaluating the measurement of informational entropy, it measures decreases in entropy when the feature value is given. This method is widely applied in applications of text categorization and classification of microarray data. On the other hand, the sequential forward floating search (SFFS) algorithm [23] is also a well-known method. The SFFS algorithm starts with an empty feature set. In each step, the best feature that satisfies some criterion function is included with the current feature set. In addition, while some feature is excluded, the SFFS algorithm also verifies the possibility of improving the criterion. Therefore the SFFS algorithm proceeds dynamically increasing and decreasing the number of features until the desired target is reached. In this paper, we compared the IG and SFFS algorithms with our method.

## 3. The Proposed Neighborhood-Relationship Feature Selection Algorithm

This section presents the proposed neighborhood-relationship feature selection (NRFS) algorithm, which consists of two main stages: adding features and eliminating features. The SFFS algorithm [23] was applied to generate an initial feature subset from the original feature set. At Stage 1 of the NRFS algorithm, the weight value of each unselected feature is calculated according to neighboring selected features. Subsequently, unselected features are added into the feature subset iteratively based on their weight value to generate a more favorable feature subset. At Stage 2 of the NRFS algorithm, a new weight value for each selected feature is calculated, and selected features are subsequently eliminated based on the new weight value. To evaluate the recognition rate of the selected feature subset, the classification method used in this study for the authentication method was the k-nearest neighbor (*k*NN) method [35,36]. The steps of the NRFS algorithm are described in detail as follows.

**Step 1: Generate the Initial Feature Subset by Using the SFFS Algorithm**

Use the SFFS algorithm to generate the initial feature subset for the NRFS algorithm.

**Step 2: Calculate the Weight Values of the Unselected Features**

**Figure 4:**
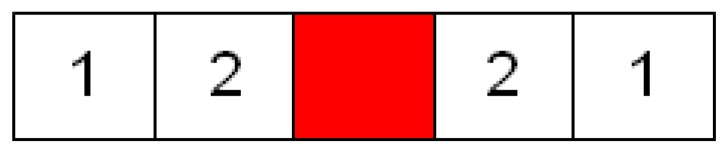
Diagram of the weight values.

**Figure 5:**
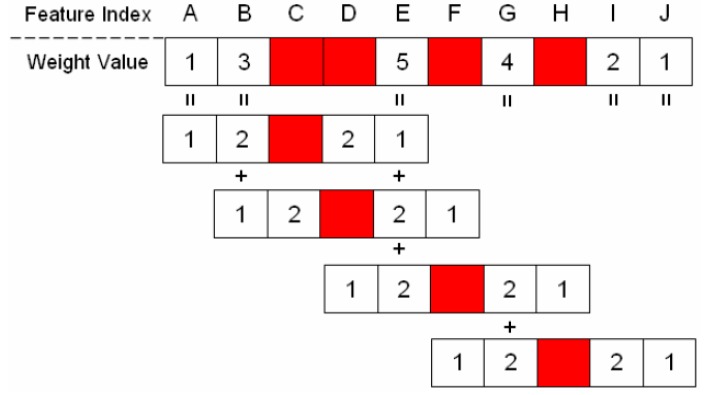
Example of how to calculate the weight values of unselected features.

To add the unselected features (the candidate features for being added) into the feature subset, the weight value of each unselected feature should first be calculated to represent the number of neighboring features that have been selected. Figure 4 shows the diagram of the weight values applied in this study. The red block in this figure denotes a feature that has been selected in the feature subset, and the neighboring unselected features are represented by the white blocks. The unselected features are assigned weight values (1 or 2) when they are located in the neighborhood of the selected feature (red block). Figure 5 shows an example calculation of the weight value of these unselected features. In this example, four features (feature indices C, D, F, and H) were selected in the feature subset. Subsequently, each unselected feature accumulated weight values that were based on neighboring selected features. As shown in Figure 5, Feature E obtained the highest weight value of 5 because it accumulated the weight values of three neighboring selected features (Features C, D, and F), whereas Feature J exhibited a weight value of only 1, which was obtained from the selected Feature H.

**Step 3: Sequentially Add a Single Feature**

In this step, sequentially add unselected features to the feature subset according to their weight values. An unselected feature with a high weight value has a high priority of being added. Table 1 shows the ranking of the unselected features in Figure 5. The example in Table 1 shows that Feature E was the first feature to be added to the feature subset. If the recognition rate of the new feature subset can be improved or remains equal to the original recognition rate, then Feature E is added to the feature subset. Subsequently, proceed to Step 2 to recalculate the weight values. Otherwise, the next unselected feature is added, depending on its ranking. If no features can be added at this step, then go to Step 4.

**Step 4: Sequentially Add Two Features**

At this step, to explore additional feature subset combinations, sequentially add two unselected features to the feature subset in each trial. Following the example in Table 1, create all possible combinations of any two unselected features and calculate the sum of their weight values. Because the amount of possible combinations of any two unselected features may be too numerous, we set a threshold T_Add_ to filter the combinations. If the weight-value sum of two selected features is larger than the threshold T_Add_ (the threshold T_Add_ was 7 in this study), then this combination of two features is a candidate combination for being added. As Table 2 shows, the weight sums of three combinations exceed the threshold T_Add_. In this scenario, sequentially add the unselected features of each combination to the feature subset. If the recognition rate of the new feature subset can be improved, or remains the same as the original recognition rate, then add the two features comprising the test combination to the feature subset. Subsequently, proceed to Step 2 to recalculate the weight values. Otherwise, add the next combination, depending on its ranking. If no features can be added at this step, go to Step 5.

**Step 5: Calculate the Weight Values of the Selected Features**

**Figure 6:**
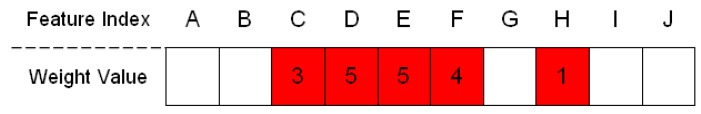
Example of how to calculate the weight values of selected features.

Before eliminating the selected features from the feature subset, recalculate the weight values of these candidate features. At this step, calculate only the weight values of the selected features for elimination in the following step. The calculation method applies the same diagram of weight values shown in Figure 4. Figure 6 shows an example of the method used to calculate the weight values of these selected features. In this example, five features (feature indices C, D, E, F, and H) were selected in the feature subset. Each selected feature exhibited a weight value that was based on neighboring selected features. For example, Feature D exhibited the highest weight value of 5 because it accumulated the weight values of three neighboring selected features (Features C, E, and F), whereas Feature H exhibited a weight value of only 1, which was obtained from the selected Feature F.

**Step 6: Sequentially Eliminate a Single Feature**

At this step, sequentially eliminate selected features from the feature subset if they have low weight values. Table 3 lists the ranking of the selected features shown in Figure 6. In the example shown in this table, Feature H was the first candidate feature for elimination from the feature subset. If the recognition rate of the new feature subset can be improved by eliminating this feature, then Feature H is removed from the feature subset. Otherwise, the next selected feature is removed, depending on its ranking, and the recognition rate is examined again. If no features can be eliminated in this step, go to Step 7.

**Step 7: Sequentially Eliminate Two Features**

Similar to Step 4, sequentially eliminate two selected features from the feature subset in each trial of this step. Following the example in Table 3, create all possible combinations of any two selected features and calculate the sum of their weight values. Subsequently, examine whether the weight-value sum of the two selected features is smaller than the threshold TDel (which was 3 in this study). In this scenario, the sum of Features C and H was smaller than the threshold TDel. Attempt to eliminate the selected Features C and H, and then examine the recognition rate. If the recognition rate of the new feature subset can be improved, eliminate the two features comprising the test combination from the feature subset and perform Step 5 to recalculate the weight values. If no other features can be eliminated at this step, then end the NRFS algorithm.

## 4. Experimental Data Generation

This section introduces the method for generating a data set of rat EEG signals and a data set of Chinese characters.

### 4.1. Rat EEG Signal Data Set

In this study, we use the same EEG signal data set in our previous study [9]. For continuous EEG monitoring, recording electrodes were chronically implanted on the skull of the rat. In this experiment, the EEG signal data is collected by only one single rat. For EEG recording, a parietal electrode was implanted on the same level of bregma. The signal was referenced to a ground electrode implanted over the cerebellum. The signal was connected to a personal computer using a connector. All of the instruments were sealed and secured to the skull with dental cement, and the skin was sutured with wound clips.

The EEG signals were recorded for 2 to 6 h at a sampling rate of 1 kHz, and were then transformed into frequency information by using FFT, as described in Section 1. The power spectrum was calculated using a 4-second window size and 1-second overlap. In general, the frequency of EEG signals is distributed in the range of 1–50 Hz. Westbrook [6] divided the observed frequencies into several groups: delta (0.5–4 Hz), theta (4–8 Hz), alpha (8–13 Hz), beta (13–20 Hz), and gamma (20–50 Hz) frequency bands. The major difference in spectrum patterns among the three states involved the delta, alpha, and gamma frequency bands.

Before constructing the classification system, an experienced expert accurately labeled the corresponding vigilance states for the data patterns. These data patterns were categorized into one of three states by examining the EEG, EMG, and locomotor behavior by using video files. Finally, a total of 810 EEG epochs were collected and labeled as data patterns in this study, in which each EEG epoch represented a 4-s period of stimulation. The data patterns were then partitioned into the training data set and testing data set. A total of 540 and 270 EEG epochs were used as the training and testing patterns, respectively. Table 4 lists the number of epochs in each state.

### 4.2. Chinese Character Data Set

**Figure 7:**
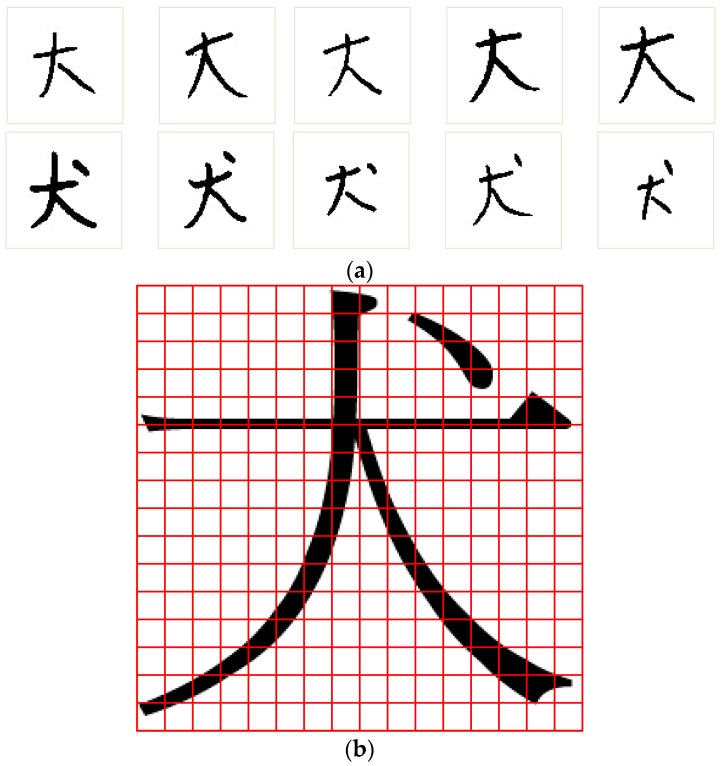
(**a**) Some examples of Chinese characters “大” and “犬”; (**b**) A character image is transfer as 256 feature vectors.

**Figure 8:**
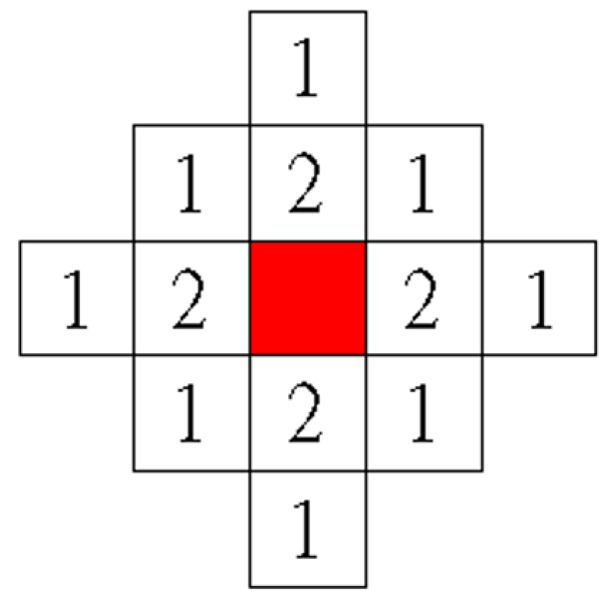
Diagram of the weight values for two-dimensional neighborhood relationship.

In this study, the proposed NRFS algorithm was applied for recognizing Chinese characters. Figure 7a shows examples of Chinese characters, “大” and “犬”, obtained from the ETL9b database [37]. The nonlinear normalization technique [38] was adopted to normalize each character image to a size of 64 × 64. Each character image was then divided into 16 × 16 subregions (Figure 7b) to generate 256 feature vectors, the values of which ranged from 0 to 16. For this application, each feature vector represents the information of a certain subregion in a character image. We believe that assuming that each feature vector has a dependent relationship with neighboring feature vectors is reasonable. However, the neighborhood relationship of a feature vector contains two-dimensional directions; therefore, the diagram of weight values in Figure 4 is not suitable for use in this situation. To solve this problem, as shown in Figure 8, a new diagram of weight values was applied for a feature vector containing a two-dimensional neighborhood relationship.

## 5. Experimental Results

Experimental simulations were conducted in this study to compare four feature-selection methods: the IG [12], sequential floating search (SFS) [23], SFFS [23], and NRFS algorithms. To extract feature subsets by using these methods, one half of the training data patterns were used to construct a classifier by applying the *k*NN classification method. The remaining data patterns in the training data set were used to estimate the accuracy rate as a criterion function of the feature selection method. After extracting the feature subset by using these feature selection algorithms, all of the training data patterns and the extracted feature subset were integrated to build a new classifier for testing and obtaining validation results from the testing data set.

### 5.1. Experiments on the Data Sets of Rat EEG Signals

In this experiment, two data sets were used to conduct the simulation. The first data set was the original data set, as described in Section 4.1. A total of 540 and 270 EEG epochs were used as the training and testing patterns, respectively. In addition, to compare the robustness of the four feature selection methods, a noisy data set was generated from the original data set. First, 20% of the data patterns were randomly chosen from the original data set. For each chosen data pattern, six features were randomly selected from the 32 features, and their values were modified. Finally, the resulting data set consisted of 20% noisy data patterns and 80% original data patterns. A comparison of the experimental results for this noisy data set can be performed to determine the robustness of the four feature selection methods.

**Experiment A-1: Original EEG Signal Data Set**

**Figure 9:**
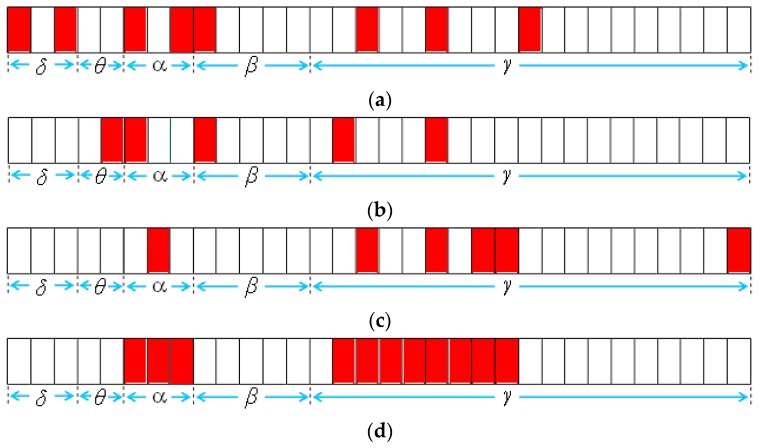
Feature vectors selected by (**a**) information gain (IG); (**b**) sequential floating search (SFS); (**c**) sequential forward floating search (SFFS); (**d**) neighborhood-relationship feature selection (NRFS).

Four feature selection algorithms were applied to extract a feature subset from the original EEG signal data set. Table 5 lists the simulation results. The NRFS algorithm extracted more features and obtained a higher accuracy rate than the IG, SFS, and SFFS algorithms did. Figure 9 shows the feature subset selected by the four algorithms. The IG, SFS, and SFFS algorithms selected eight, five, and six feature vectors from the original data set, respectively, as shown in Figure 9a–c. The NRFS algorithm selected 11 feature vectors (Figure 9d), and the accuracy rate was higher than that of the other algorithms. The feature vectors selected by the NRFS algorithm were concentrated in two main regions. The frequency bands of the selected feature vectors were at 8–12.8 Hz and 22.4–35.2 Hz for these two main regions. The 8–12.8 Hz frequency band was identified as the alpha band, and the 22.4–35.2 Hz frequency band was identified as the lower gamma band. The simulation result of applying the NRFS algorithm is in part agreement with that obtained by Westbrook [6] and Louis [8]. More discussions are given in Section 6.

**Experiment A-2: Noisy EEG Signal Data Set**

**Figure 10:**
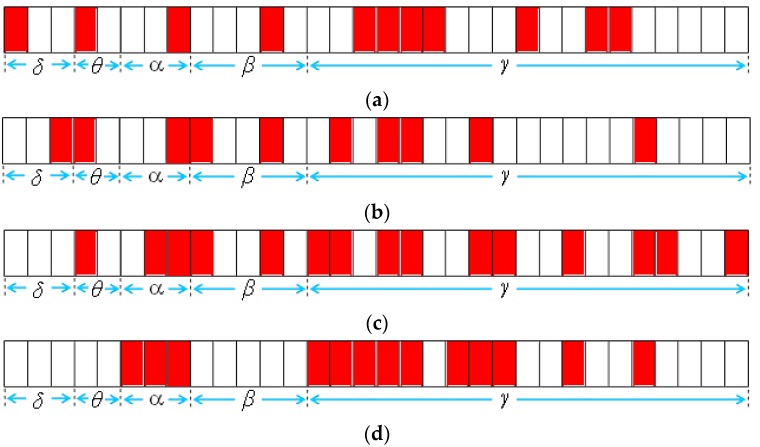
Feature vectors selected by (**a**) IG; (**b**) SFS; (**c**) SFFS; (**d**) NRFS.

A noisy data set was generated from the original data set to compare the robustness of the four feature selection methods. The noisy EEG signal data set contained 20% noisy data patterns and 80% original data patterns. Table 6 lists the simulation results, and Figure 10 shows the feature subset selected by the four algorithms. Once again, the NRFS algorithm obtained a higher accuracy rate than the IG, SFS, and SFFS algorithms did. Although the data set contained noisy data patterns, the NRFS algorithm could extract features that were comparable to those identified in Experiment A-1 (Figure 9d). However, the features extracted by the IG, SFS, and SFFS algorithms differed considerably from the feature subset obtained in Experiment A-1 (Figure 9a–c). This experiment demonstrated that the proposed algorithm is robust when the data set contains noisy data.

### 5.2. Experiments on the Data Sets of Chinese Characters

**Figure 11:**
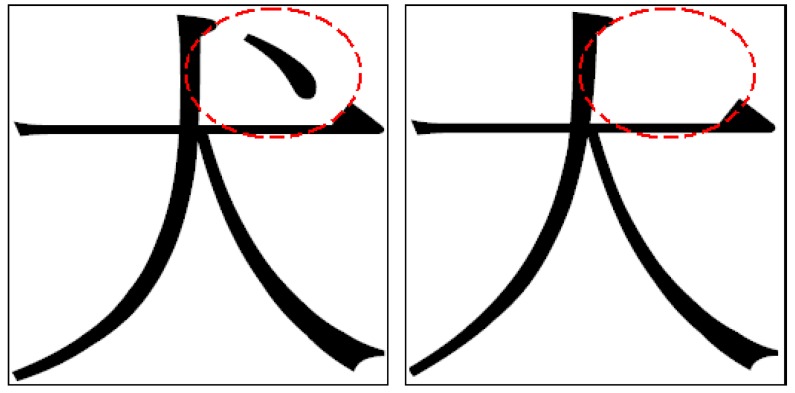
The crucial area for classifying the characters “犬” and “大”.

In this experiment, two data sets were used for conducting the simulation. In the first data set, two classes of Chinese characters were used, “犬” and “大”, and each class contained 200 character images. As shown in Figure 11, the top-right area of the character image was the crucial area for classifying the characters “犬” and “大” (Figure 11). In the data set, the number of training and testing patterns were 266 and 134, respectively. The same process described in Section 5.1 was applied, and a noisy data set was generated from the original data set of Chinese characters. The noisy data set consisted of 20% noisy data patterns and 80% original data patterns. A comparison of the experimental results obtained for this noisy data set was conducted to determine the robustness of the three feature selection methods.

**Experiment B-1: Original Data Set of Chinese Characters**

**Figure 12:**
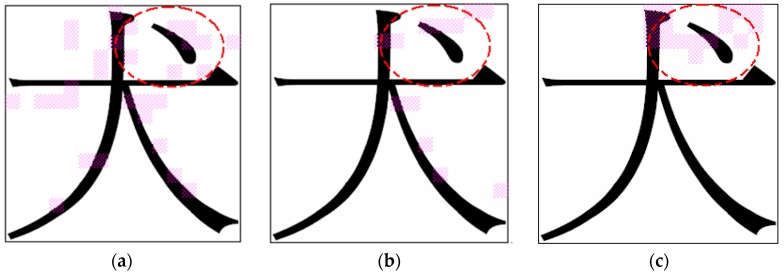
Feature vectors selected by (**a**) IG; (**b**) SFFS; (**c**) NRFS.

In the experiment, the IG, SFFS, and NRFS algorithms were applied to extract a feature subset from the original data set of Chinese characters. For the recognition of Chinese characters, the two-dimensional diagram of weight values (Figure 8) was applied in the NRFS algorithm. Table 7 lists the simulation results. The NRFS algorithm extracted more features and obtained a higher accuracy rate than the IG and SFFS algorithms did. Figure 12 shows the feature subset selected by the three algorithms. If a feature vector is selected by an algorithm, then the corresponding subregion of the character image is labeled in pink as shown in Figure 12. In Figure 12c, the NRFS algorithm selected more feature vectors in the top-right area of the character image, which was the crucial area used to classify the characters “犬” and “大”. However, in Figure 12a,b, the subregions selected by IG and SFFS were spread across various locations. Consequently, locating the crucial area for classifying the characters “犬” and “大” was difficult. The simulation result indicates that the NRFS performed favorably when the features exhibited a two-dimensional neighborhood relationship.

**Experiment B-2: Noisy Data Set of Chinese Characters**

**Figure 13:**
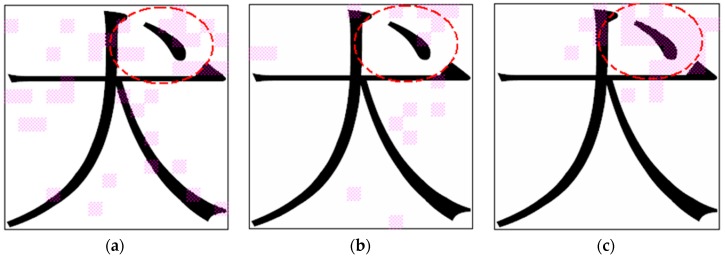
Feature vectors selected by (**a**) IG; (**b**) SFFS; (**c**) NRFS.

In this experiment, a noisy data set was generated from the original data set to compare the robustness of the three feature selection methods. Table 8 lists the simulation results, and Figure 13 shows the feature subset selected by the three algorithms. Although the data set contained noisy data patterns, the NRFS algorithm obtained a higher accuracy rate than the IG and SFFS algorithms did. In addition, the NRFS algorithm extracted feature vectors that were comparable to those identified in Experiment B-1 (Figure 12c). However, the features extracted by the IG and SFFS algorithms also differed considerably from the feature subset obtained in Experiment B-1 (Figure 12a,b). Once again, this experiment demonstrated that the NRFS algorithm is robust when the data set contains noisy data.

## 6. Discussion

In Experiment A-1, the NRFS algorithm was applied to select the critical frequency bands in classifying three vigilance stages of rats. Finally, the frequency bands of the selected feature vectors were at alpha band (8–12.8 Hz) and lower gamma band (22.4–35.2 Hz) for these two main regions. According to research results of Westbrook [6] and Louis *et al.* [8], they considered that the major difference of spectrum pattern in the delta (0.5–4 Hz), alpha (8–13 Hz), and gamma (20–50 Hz) bands were the key features for classifying three vigilance stages. In addition, they also suggest distinguishing active awake from quiet awake by observing high EMG activity. Compared with their research results, the NRFS algorithm selected feature vectors at alpha and gamma bands, however, it did not select any feature vector at delta band. To explain this result, we have following observations.

1. The proposed NRFS algorithm uses the SFFS algorithm to generate the initial feature subset. If the initial feature subset does not include any feature vector in the delta band, the NRFS algorithm usually cannot have a change to extract any feature vector from the delta band. This means the performance of the NRFS algorithm is sensitive to its initial feature subset.
2. By further examining the data patterns in the EEG signal dataset, in the REM state, the data patterns have high amplitude in the lower gamma band. Additionally, in the lower gamma band, the data patterns show median amplitude for the AW state and show lower amplitude for the SWS state, respectively. It means that the feature vectors at the low gamma band can be the key features to identify three vigilance stages. To our collected EEG-signal data set, when the NRFS algorithm selects enough feature vectors from the lower gamma band into the feature subset, this feature subset can usually achieve a high accuracy rate. This situation also reduces the possibility to select feature vectors in the delta band for the NRFS algorithm.

Although the NRFS algorithm achieves good performance in identifying the crucial frequency band for classifying vigilance stages, however, in this paper, the EEG-signal data set was collected by a single rat. As a result, the generalizability of the NRFS algorithm is limited. In the future, we would collect more EEG-signal data sets to further examine the performance of the NRFS algorithm.

## 7. Conclusions

This study proposes using the NRFS algorithm to identify crucial frequency bands for classifying the vigilance states of rats, and for locating crucial areas in a character image for recognizing Chinese characters. The proposed algorithm adopts the neighborhood-relationship concept when adding and eliminating candidate features. The experimental results of this study indicated that the NRFS algorithm achieves satisfactory accuracy and demonstrates robustness when analyzing noisy data. Furthermore, the NRFS algorithm identifies crucial frequency bands and produces interesting results akin to those that have been obtained by biological researchers. In addition, the simulation results of Chinese character recognition indicate that the NRFS performs favorably when the features exhibit a two-dimensional neighborhood relationship.

## Figures and Tables

**Table 1:** Weight value and ranking of unselected features of Figure 5.

Feature Index	Feature Subset	Weight Value	Ranking of Adding a Single Feature
A		1	5
B		3	3
C	Chosen		
D	Chosen		
E		5	1
F	Chosen		
G		4	2
H	Chosen		
I		2	4
J		1	5

**Table 2:** Weight sum of two unselected features and their ranking.

The Combination of Two Unselected Features	Sum of Weight Values	Ranking of Adding Two Features
Feature E and Feature G	9	1
Feature E and Feature B	8	2
Feature G and Feature B	7	3

**Table 3:** The weight values and ranking of selected features of Figure 6.

Feature Index	Feature Subset	Weight Value	Eliminating a Single Feature
A			
B			
C	Chosen	3	2
D	Chosen	5	4
E	Chosen	5	4
F	Chosen	4	3
G			
H	Chosen	1	1
I			
J			

**Table 4:** Number of epochs in each state.

Vigilance State	Number of Training Epochs	Number of Testing Epochs
REM (rapid eye movement)	56	41
SWS (slow wave sleep)	248	155
AW (awake)	236	74
Total Number	540	270

**Table 5:** Experimental results of original data set of EEG signals.

	IG	SFS	SFFS	NRFS
**Number of Selected Feature Vectors**	8	5	6	11
**Accuracy of Testing Data (%)**	89.26	91.85	93.33	94.81
**Computational Time (s)**	1.2	0.6	2.8	42.5

**Table 6:** Experimental results of noisy data set of EEG signals.

	IG	SFS	SFFS	NRFS
**Number of Selected Feature Vectors**	11	10	15	13
**Accuracy of Testing Data (%)**	85.92	84.81	88.51	92.60
**Computational Time (s)**	1.3	0.8	3.1	68.8

**Table 7:** Experimental results of original data set of Chinese characters.

	IG	SFFS	NRFS
**Number of Selected Feature Vectors**	30	12	16
**Accuracy of Testing Data (%)**	91.04	95.52	97.01
**Computational Time (s)**	11	25	406

**Table 8:** Experimental results of noisy data set of Chinese characters.

	IG	SFFS	NRFS
**Number of Selected Feature Vectors**	38	16	30
**Accuracy of Testing Data (%)**	90.03	94.02	96.27
**Computational Time (s)**	13	29	506
